# Leading Topics in Twitter Discourse on JUUL and Puff Bar Products: Content Analysis

**DOI:** 10.2196/26510

**Published:** 2021-07-19

**Authors:** Jon-Patrick Allem, Allison Dormanesh, Anuja Majmundar, Vanessa Rivera, Maya Chu, Jennifer B Unger, Tess Boley Cruz

**Affiliations:** 1 Department of Preventive Medicine Keck School of Medicine University of Southern California Los Angeles, CA United States; 2 Department of Surveillance and Health Equity Science American Cancer Society Washington, DC United States

**Keywords:** electronic cigarettes, JUUL, public health, Puff Bar, social media, Twitter, infodemiology

## Abstract

**Background:**

In response to the recent government restrictions, flavored JUUL products, which are rechargeable closed-system electronic cigarettes (e-cigarettes), are no longer available for sale. However, disposable closed-system products such as the flavored Puff Bar e-cigarette continues to be available. If e-cigarette consumers simply switch between products during the current government restrictions limited to 1 type of product over another, then such restrictions would be less effective. A step forward in this line of research is to understand how the public discusses these products by examining discourse referencing both Puff Bar and JUUL in the same conversation. Twitter data provide ample opportunity to capture such early trends that could be used to help public health researchers stay abreast of the rapidly changing e-cigarette marketplace.

**Objective:**

The goal of this study was to examine public discourse referencing both Puff Bar and JUUL products in the same conversation on Twitter.

**Methods:**

We collected data from Twitter’s streaming application programming interface between July 16, 2019, and August 29, 2020, which included both “Puff Bar” and “JUUL” (n=2632). We then used an inductive approach to become familiar with the data and generate a codebook to identify common themes. Saturation was determined to be reached with 10 themes.

**Results:**

Posts often mentioned flavors, dual use, design features, youth use, health risks, switching 1 product for the other, price, confusion over the differences between products, longevity of the products, and nicotine concentration.

**Conclusions:**

On examining the public’s conversations about Puff Bar and JUUL products on Twitter, having described themes in posts, this study aimed to help the tobacco control community stay informed about 2 popular e-cigarette products with different device features, which can be potentially substituted for one another. Future health communication campaigns may consider targeting the health consequences of using multiple e-cigarette products or dual use to reduce exposure to high levels of nicotine among younger populations.

## Introduction

Electronic cigarettes (e-cigarettes) are popular in the United States [1,2]. In February 2020, the US Food and Drug Administration (FDA) restricted flavored closed-system cartridge e-cigarettes (eg, JUUL), with the exception of tobacco and menthol flavors, in an effort to discourage their use among the youth [3,4]. These restrictions did not apply to relatively new disposable (nonrefillable) e-cigarettes [2]. For instance, Puff Bar offers disposable nicotine salt–based products (also found in JUUL products), in over 20 flavors such as pink lemonade. Congressional lawmakers petitioned the FDA to restrict Puff Bar, arguing that they were the fastest growing e-cigarette brand, replacing JUUL as the e-cigarette of choice among the youth [5]. The FDA sent warning letters to Puff Bar instructing them to remove their flavored disposable e-cigarettes from the market because the latter did not have the required premarket authorization [6]. In response, Puff Bar briefly stopped selling their products through their official website, but their products were always available for purchase from third-party websites.

Recent evidence from Google search trends suggests that public interest in Puff Bars surged immediately after the FDA announced a restriction on flavored e-cigarettes [7]. If consumers simply switch to disposable products during the present restriction on flavored closed-system products, then such restrictions would be less effective. However, it is unclear whether the public views disposable and reusable e-cigarettes as ideal substitutes. In other words, public discourse discussing the product features and user experiences with JUUL and Puff Bar is understudied. A step forward in this line of research is to describe public discourse referencing both Puff Bar and JUUL products in the same conversation.

This study utilized Twitter data to examine public conversations about Puff Bar and JUUL products during a time of change in the e-cigarette marketplace. Twitter has previously been used to describe the context of e-cigarette–related attitudes and behaviors in a way that offers direct insights on user experience, including preferred design features and flavor preferences [8]. By examining the public’s conversations about Puff Bar and JUUL products on Twitter, having described themes in posts, this study aims to help the tobacco control community stay informed about 2 popular e-cigarette products with different device features, which can be potentially substituted for one another. Our findings may inform FDA policy targets and communication strategies in the future.

## Methods

Posts containing both terms “Puff Bar” and “JUUL” were collected from July 16, 2019, to August 29, 2020, from Twitter’s streaming application programming interface (n=2632). Similar to prior studies [8], retweets were removed so that each observation could be treated as an independent observation (n=1577). Two trained researchers manually coded tweets into themes, using an inductive approach. The goal of this approach was to condense the raw text-based data into a summary format and report the underlying patterns that were evident in the data. The unit of analysis was the text. Saturation was determined to be reached with 10 themes.

The codebook (Table 1) consisted of the following themes: (1) device features, including mentions of hardware, product features, specifications, and product quality; (2) flavors, including mentions of flavors offered by each brand or enjoyed by the consumer; (3) longevity, including mentions of how long a Puff Bar or JUUL product lasts, such as the duration and number of puffs; (4) price, including mentions of monetary amounts or affordability of JUUL and Puff Bar products; (5) youth use, including mentions of youth (aged under 21 years) and mentions of children, youth, or teenagers using a Puff Bar or JUUL product or other e-cigarette products during school time or in school premises; (6) switching, including mentions of substituting 1 product with the other; (7) dual use, including mentions of using both Puff Bar and JUUL products; (8) nicotine concentration, including mentions of nicotine concentration or nicotine salt levels; (9) health risks, including mentions of Puff Bar products being more harmful than other e-cigarettes (eg, JUUL e-cigarettes) or vice versa, and of negative health consequences of vaping; and (10) confusion, including mentions of confusion over the differences between the features of Puff Bar and JUUL products. Posts were segregated into multiple themes.

To establish interrater reliability, coders analyzed a subsample of posts (n=300), with agreement ranging from 84% to 97%. The lead author served as the arbitrator and resolved disagreements. Descriptive statistics were reported in a confusion matrix to show the prevalence of each theme as well as theme cooccurrence in a single post. Data collection processes relied on publicly available data and adhered to Twitter’s terms and conditions, terms of use, and privacy policies. The protocol was approved by the university’s institutional review board (protocol# HS-18-00697).

**Table 1:** Definitions for each theme and example paraphrased posts.

Theme	Definition	Paraphrased post
Device features	Mentions of hardware (eg, disposable or reusable battery), product features (eg, color of the device), specifications, and quality of the product.	Puff Bar is a disposable, prefilled, and precharged vape, but JUUL has disposable pods and a reusable battery.
Flavors	Mentions of flavors (eg, specific fruit, sweet, savory, candy, alcohol, coffee, tobacco, menthol, or mint) offered by each brand or enjoyed by the consumer.	This banana-flavored Puff Bar e-cigarette tastes amazing compared to a tobacco-flavored JUUL e-cigarette.
Longevity	Mentions of how long a Puff Bar or JUUL product lasts, including the duration and number of puffs.	This Puff Bar only lasts 1 day, but I cannot afford a pack of JUUL pods that last 2 weeks.
Price	Mentions of monetary amounts or affordability of JUUL and Puff Bar products.	Puff Bar [products] cost only US $8 and are cheaper than JUUL [products].
Youth use	Mentions of youth (aged under 21 years) and mentions of children, youth, or teenagers using a Puff Bar or JUUL product or other e-cigarette products during school time or in school premises. Posts may also raise concerns over youth use of vaping products in general.	[I] found a JUUL [e-cigarette] in the high school bathroom in the morning and a Puff Bar [e-cigarette] again later.
Switching	Mentions of quitting 1 product for the other.	I quit [using] JUUL [e-cigarettes], but now I just use Puff Bar [e-cigarettes] every day.
Dual use	Mentions of using both Puff Bar and JUUL products.	[I am] hitting my JUUL [e-cigarette] for breakfast and my pink lemonade Puff Bar [e-cigarette] for dinner.
Nicotine concentration	Mentions of nicotine concentration or nicotine salt levels.	I can get just as much nicotine from Puff Bar [e-cigarettes] as my JUUL [e-cigarette] with even higher nicotine delivery.
Health risks	Mentions of Puff Bar being more harmful than other e-cigarettes (eg, JUUL) or vice versa, and of negative health consequences of Puff Bar products. This may include mentions of people harming themselves by using JUUL or Puff Bar products.	Puff Bar and JUUL [e-cigarettes] made my chest hurt so bad, but I still use my vape.
Confusion	Mentions of confusion over the differences between Puff Bar products and other e-cigarettes (eg, JUUL).	She was holding a Puff Bar or maybe it was a disposable JUUL [e-cigarette]?

## Results

The most prominent topic was “flavors” (n=311 of 1577 posts, 19.72%), followed by “dual use” (n=254, 16.11%), “device features” (n=230, 14.58%), and “youth use” (n=219, 13.89%) (Table 2). These were followed by “health risks” (n=130, 8.24%), “switching” (n=105, 6.66%), “price” (n=77, 4.88%), “confusion” (n=49, 3.11%), “longevity” (n=47, 2.98%), and “nicotine concentration” (n=42, 2.66%). The most common cooccurring themes in a single post were “youth use” and “device features” (n=70, 4.44%)*,* followed by “device features” and “flavors” (n=67, 4.25%) and “youth” and “flavors” (n=61, 3.87%).

**Table 2:** Prevalence of themes^a^.

Themes	Flavors	Dual use	Device features	Youth use	Health risks	Switching	Price	Confusion	Longevity	Nicotine concentration
Nicotine concentration	10 (0.63)	1 (0.06)	17 (1.08)	16 (1.01)	4 (0.25)	2 (0.13)	4 (0.25)	0 (0.00)	1 (0.06)	42 (2.66)
Longevity	11 (0.70)	10 (0.63)	7 (0.44)	3 (0.19)	2 (0.13)	4 (0.25)	14 (0.89)	0 (0.00)	47 (2.98)	
Confusion	10 (0.63)	0 (0.00)	3 (0.19)	2 (0.13)	2 (0.13)	0 (0.00)	0 (0.00)	49 (3.11)		
Price	21 (1.33)	4 (0.25)	23 (1.46)	5 (0.32)	2 (0.13)	4 (0.25)	77 (4.88)			
Switching	28 (1.78)	4 (0.25)	5 (0.32)	14 (0.89)	2 (0.13)	105 (6.66)				
Health risks	13 (0.82)	6 (0.38)	9 (0.57)	17 (1.08)	130 (8.24)					
Youth use	61 (3.87)	24 (1.52)	70 (4.44)	219 (13.89)						
Device features	67 (4.25)	17 (1.08)	230 (14.58)							
Dual use	31 (1.97)	254 (16.11)								
Flavors	311 (19.72)									

^a^The diagonal line indicates the prevalence of the 10 topics identified. The off-diagonal lines indicate topic overlap. All values are presented as numbers and percentages in parentheses.

## Discussion

### Principal Findings

This study provides a summary of public Twitter posts collected over the course of a 13-month period, which includes mentions of both “Puff Bar,” a disposable e-cigarette, and “JUUL,” a reusable closed-system cartridge e-cigarette. Posts often mentioned flavors, dual use, device features, youth use, health risks, switching 1 product for the other, price, confusion over the differences between products, longevity of products, and nicotine concentration. Theme cooccurrence in a single post was also examined.

“Flavors” was the most common theme in this study, while “flavors” and “device features” represented the second-most common theme cooccurrence in a single post. Prior studies that examined tobacco-related (eg, hookah or little cigars) conversations on Twitter have identified similar themes [9,10]. The FDA has previously taken action to reduce the appeal of e-cigarettes among the youth by removing flavored products. The FDA recently sent warning letters to 10 companies, including Puff Bar, to remove their flavored disposable e-cigarettes from the market because they do not have the required premarket authorization [6]. Puff Bar’s compliance with this request and FDA’s enforcement will dictate whether their products will be less readily available for purchase.

“Device features” was a predominant theme in this study, while “youth use” and “device features” represented the most common theme cooccurrence in a single post. Previous studies have suggested that product features create lasting psychological, sensory, and behavioral responses among consumers, which may translate to appeal for these products [11]. Additionally, consumers satiate less when similar products are presented as distinct subcategories [12]. In other words, although both JUUL and Puff Bar products are e-cigarettes, consumers may be attracted to using Puff Bar if they perceive it as an e-cigarette product with unique features (such as disposability). Identifying and regulating youth-appealing device features (eg, age restrictions on the purchase of disposable products that are youth-appealing and mandating plain device colors to address attractive designs) may facilitate more effective tobacco control efforts.

Dual use of JUUL and Puff Bar products raises concerns about inadvertent exposure to high levels of nicotine among the youth. A recent study [13] suggests that young adults find it difficult to understand nicotine concentration. When consumers are familiar with both products displaying nicotine levels as mg/mL and percentages, they are more likely to have a correct understanding of nicotine strength [13]. Currently, the official JUUL website and packaging labels list nicotine concentration as percentage values. Similarly, the official Puff Bar website and packaging labels list nicotine concentration as percentage levels; however, this metric appears differently on other retail platforms. As such, regulations standardizing the labeling of nicotine concentration on web-based retail platforms and on product packaging may facilitate consumer awareness. Future health communication campaigns may also consider targeting the health consequences of using multiple e-cigarette products to reduce the dual use of e-cigarette products.

Our findings suggest that there was some level of confusion over the differences or similarities among Puff Bar, JUUL, and other e-cigarettes. Confusion may render Twitter users vulnerable to inaccurate information about the health effects of these products and likely to misjudge these products’ potential relative health risks. A prior study [8] reported that the phrase “What is JUUL?” appeared commonly on Twitter in 2017. Public health communication campaigns need to discuss the health risks of popular emerging products including Puff Bar e-cigarettes as they become increasingly available in the market, to keep parents, educators, and clinicians well-informed of the rapidly evolving e-cigarette marketplace.

Prior studies suggest that marketplaces where consumers can switch to other products in a short period, with limited effort or at a lower price, typically allow easy entry of newer products and facilitate rapid consumer migration to newer products [14,15]. While both JUUL and Puff Bar products contain nicotine salts, Puff Bar potentially facilitates easy switching, given these are single-use products available at a lower cost per unit [16]. Additionally, since Puff Bar is a relatively new e-cigarette brand and its technology could be replicated by other companies easily [17], consumers in the e-cigarette marketplace may transition to other unregulated products. Regulations that create barriers for the entry of similar products and deincentivize consumers to switch to other flavored products are crucial. Currently, flavor restrictions have been applied narrowly to specific product lines, which may make it easier for new products such as those of Puff Bar to circumvent regulations and normalize switching behavior for vape products among consumers.

### Limitations

This study was limited to the analysis of discussions on 2 e-cigarette brands, Puff Bar and JUUL, and may not pertain to other e-cigarette brands. However, Puff Barr and JUUL e-cigarettes appear to represent the market leaders for disposable e-cigarettes and reusable closed-system cartridge e-cigarettes, respectively. This study only collected tweets that mentioned the 2 products (Puff Bar and JUUL) in the same post. This decision may have excluded select posts that may have been relevant to our study. This study focused on Twitter posts, and our findings may not generalize to other social media platforms. The posts in this study were collected within a 13-month period and may not extend to other time periods. Data collection relied on Twitter’s streaming application programming interface, which prevented the collection of posts from private accounts. Our findings may not be generalizable to all Twitter users or to the population of the United States.

### Conclusions

Our findings may offer a point of departure for understanding the public’s understanding of and experience with disposable and reusable closed-system cartridge e-cigarettes. Future studies should identify the features of youth-appealing e-cigarette devices to inform more targeted tobacco regulations. Studies should focus on effective communication strategies to raise awareness about known health risks pertaining to dual use and product substitution or switching and about new tobacco products among parents, educators, and vulnerable communities. Comprehensive tobacco regulations may include extending ongoing and upcoming restrictions prospectively to existing and future products, to prevent new products from circumventing current regulations. Regulations mandating standardized labeling of nicotine concentration on web-based platforms may help address health risks from nicotine overdose when consumers switch products. Social media surveillance can help capture new products emerging in the marketplace, such as Puff Bar products, and monitor the web-based marketplace to prevent the sales of nonregulated flavored products.

## Abbreviations

e-cigarette: electronic cigarette
FDA: US Food and Drug Administration
